# Factors Associated with Adjuvant Radiotherapy Use After Simpson Grade I Resection of WHO Grade 2 Meningioma: A Single-Center Retrospective Cohort Study

**DOI:** 10.3390/diagnostics16203286

**Published:** 2026-10-10

**Authors:** Yasar Unsal, Mehmet Ozgur Ozates, Recai Kulak, Erva Eser, Atilla Kazanci

**Affiliations:** 1 Department of Neurosurgery, Ankara Bilkent City Hospital, Ankara 06800, Türkiye; 2 Department of Neurosurgery, Ankara Yıldırım Beyazıt University, Ankara 06760, Türkiye; 3 Department of Biostatistics, Kırıkkale University, Kırıkkale 71450, Türkiye

**Keywords:** WHO grade 2 meningioma, adjuvant radiotherapy, Ki-67, mitotic index, Simpson grade, treatment allocation, real-world practice

## Abstract

**Background:** Adjuvant radiotherapy (RT) after gross-total resection of World Health Organization (WHO) grade 2 meningioma remains an individualized decision in the absence of definitive randomized evidence. We aimed to identify the clinical, radiological, and pathological factors associated with observed adjuvant RT allocation after Simpson grade I resection and to evaluate the discriminatory performance of proliferative biomarkers in real-world multidisciplinary practice. **Methods:** This single-center, retrospective cohort included 65 patients, of whom 56 undergoing Simpson grade I resection constituted the primary analytic cohort. Associations with observed RT allocation were evaluated using Firth-penalized logistic regression. Ki-67 and mitotic-index discrimination was summarized using receiver operating characteristic (ROC) analysis, and Ki-67 threshold stability was assessed in 5000 bootstrap resamples. Brain invasion was evaluated in a documented-case sensitivity analysis. **Results:** Adjuvant RT was administered to 24 of 56 patients (42.9%). In models adjusted for age, sex, neutrophil-to-lymphocyte ratio, and tumor size, each 5-percentage-point increase in Ki-67 was associated with higher odds of RT allocation (OR 7.10, 95% CI 2.20–22.94; *p* = 0.001), as was each increase of 5 mitoses per 10 high-power fields in mitotic index (OR 5.81, 95% CI 2.01–16.75; *p* = 0.001). Ki-67 showed greater apparent discrimination than mitotic index (area under the curve [AUC] 0.925 vs. 0.801; DeLong *p* = 0.008). The apparent Youden-optimal Ki-67 threshold was 14%, but thresholds of 12%, 14%, and 15% were selected in 33.4%, 41.9%, and 23.1% of bootstrap samples. Among 44 patients with documented brain-invasion status, Ki-67 remained associated with RT allocation after adjustment for brain invasion (OR 4.53, 95% CI 1.77–11.63; *p* = 0.002). **Conclusions:** Ki-67 and mitotic index were associated with observed RT allocation, but both markers were already available to the tumor board. These findings describe institutional treatment-allocation patterns and do not establish RT benefit or validate a Ki-67 threshold for recommending treatment.

## 1. Introduction

Meningiomas are the most common primary intracranial tumors in adults, and World Health Organization (WHO) grade 2 meningiomas constitute a clinically important subgroup because of their higher risk of recurrence [1,2]. Surgical resection remains the cornerstone of treatment, with the extent of resection traditionally classified according to the Simpson grading system [2,3]. Although Simpson grades I–III resections are generally considered gross-total resection (GTR), WHO grade 2 meningiomas may recur despite complete tumor removal [4]. Adjuvant radiotherapy (RT) is generally considered for subtotal resection, recurrent disease, or other high-risk situations; however, its routine use after GTR remains controversial [2].

Prospective phase II studies have demonstrated favorable tumor control after postoperative RT in patients with completely resected WHO grade 2 meningioma [5,6]. However, these studies did not provide randomized comparisons between adjuvant RT and postoperative surveillance. The ROAM/EORTC-1308 trial was designed to address this uncertainty by comparing early adjuvant RT with active MRI surveillance after complete resection of atypical meningioma [4,7]. Until randomized evidence becomes available, postoperative management after GTR remains individualized, and treatment decisions are frequently made within multidisciplinary tumor boards.

In clinical practice, RT selection may incorporate surgical, anatomical, pathological, and patient-related factors. Among these, proliferative markers are particularly relevant to postoperative risk assessment. The 2021 WHO classification incorporates mitotic activity and brain invasion into the diagnostic criteria for grade 2 meningioma [8]. The Ki-67 labeling index provides complementary information on proliferative activity and has repeatedly been associated with meningioma recurrence, although its clinical interpretation is limited by variability in measurement and the absence of universally accepted thresholds [9,10,11]. Mitotic count has likewise been associated with recurrence and is directly incorporated into histopathological grading [12,13]. Nevertheless, the relative influence of these proliferative markers on actual postoperative RT selection after complete resection remains poorly characterized.

Characterizing the determinants of RT allocation is clinically relevant because treatment assignment in retrospective cohorts is inherently nonrandom and may result in substantial confounding by indication. Extent of resection represents a particular source of such confounding, as patients with Simpson grades II–III resection may be more likely to receive postoperative RT than those with Simpson grade I resection. Restricting the analysis to a homogeneous Simpson grade I population therefore provides an opportunity to examine treatment-selection patterns independently of variation in extent of resection.

Accordingly, we examined clinical, radiological, and pathological factors associated with observed adjuvant RT use after Simpson grade I resection of WHO grade 2 meningioma. Because Ki-67 and mitotic index were available to the multidisciplinary tumor board, their associations with RT use describe how recorded pathological findings aligned with subsequent treatment allocation. This exploratory study was designed to characterize institutional allocation patterns, not to determine treatment benefit or establish a biomarker threshold for recommending RT.

## 2. Materials and Methods

### 2.1. Study Design and Ethical Approval

This retrospective, single-center cohort study was conducted at Ankara Bilkent City Hospital in accordance with the Declaration of Helsinki. The study protocol was approved by the Institutional Ethics Committee of Ankara Bilkent City Hospital (approval no. TABED 2-25-1773; 21 January 2025). The ethics committee waived the requirement for written informed consent owing to the retrospective study design. Consecutive patients treated between January 2021 and December 2024 were retrospectively screened.

### 2.2. Patient Population and Eligibility Criteria

Consecutive adult patients who underwent surgical resection for meningioma were screened for eligibility. All cases underwent retrospective histopathological reassessment according to the 2021 WHO Classification of Central Nervous System Tumours, irrespective of the classification used at the initial diagnosis. The eligible cohort comprised 65 patients confirmed as WHO grade 2 on reassessment, including 56 patients with Simpson grade I resection who constituted the primary analytic cohort. Inclusion criteria were as follows: (1) age ≥18 years at the time of surgery; and (2) gross-total resection, defined as Simpson grade I, II, or III resection. Exclusion criteria were as follows: (1) subtotal resection, defined as Simpson grade IV or V resection; (2) diagnosis of neurofibromatosis type 2; and (3) insufficient postoperative clinical or treatment records to determine the initial adjuvant management strategy.

Of the 65 eligible patients meeting these criteria, 56 underwent Simpson grade I resection and constituted the primary analytic cohort for the treatment-decision analysis. The remaining nine patients underwent Simpson grade II or III resection. Because extent of resection within the gross-total category was strongly associated with adjuvant RT use, and because all patients with Simpson grades II–III resection in this cohort received RT, these nine patients were described separately and excluded from the multivariable decision models to avoid confounding by extent of resection and complete separation.

### 2.3. Extent of Resection and Simpson Grade Assessment

The Simpson grade was assessed retrospectively by two neurosurgeons (Y.U. and M.O.O.) through joint review of the operative reports and available postoperative MRI studies, with the final grade assigned by consensus. Both reviewers were unaware of the adjuvant RT decision during this assessment. Operative reports were used to establish the extent of tumor removal and the management of the dural attachment and involved bone, while postoperative MRI was used to corroborate the presence or absence of residual tumor. No discrepancies between the operative reports and the available postoperative MRI findings were identified. Simpson grade I was defined as complete tumor removal with resection of the involved dura and abnormal bone when present. Simpson grade II was defined as complete tumor removal with coagulation of the dural attachment. Simpson grade III was defined as complete tumor removal without resection or coagulation of the dural attachment or extradural extension. Although Simpson grades I–III resections were collectively considered gross-total resection for eligibility, the specific Simpson grade was recorded separately for each patient.

### 2.4. Histopathological and Immunohistochemical Evaluation

All tumor specimens were fixed in 10% neutral-buffered formalin, paraffin-embedded, and cut into 4 μm sections. Hematoxylin and eosin-stained sections were used for histopathological diagnosis and mitotic index assessment. The mitotic index was determined by counting the number of mitotic figures per 10 high-power fields (HPFs), using a 40× objective (400× total magnification; field area 0.196 mm^2^). Mitotic counts were performed in the most cellular tumor areas (hot spots); atypical, apoptotic, or questionable figures were excluded from the final count.

The Ki-67 labeling index was assessed by immunohistochemistry using the anti-Ki-67 monoclonal antibody clone MIB-1. After antigen retrieval, sections were incubated with the primary antibody followed by a horseradish peroxidase-conjugated secondary antibody. The Ki-67 labeling index was calculated as the percentage of positively stained tumor nuclei among at least 1000 tumor cells in the areas with the highest staining density; nuclear staining of any intensity was considered positive.

All histopathological evaluations, including mitotic index and Ki-67 labeling index, were performed independently by two neuropathologists blinded to clinical outcomes, postoperative treatment allocation, and follow-up data. Interobserver agreement was assessed in a randomly selected subset of 20 cases and was substantial for mitotic index (intraclass correlation coefficient [ICC], 0.82) and good for Ki-67 labeling index (ICC, 0.76). In cases of disagreement, defined as a >10% relative difference for Ki-67 or a difference of >2 mitoses/10 HPF for mitotic index, consensus was reached through joint slide re-evaluation. The agreed Ki-67 value was documented in the final routine pathology report. For this study, investigators extracted the Ki-67 values from these reports, which were available to the multidisciplinary tumor board when postoperative treatment decisions were made.

### 2.5. Multidisciplinary Postoperative Decision-Making Process

Postoperative management was discussed by a multidisciplinary tumor board with representatives from neurosurgery, radiation oncology, neuroradiology, and neuropathology. Decisions regarding adjuvant RT were based on an individualized assessment of surgical, radiological, histopathological, and patient-related factors rather than a single biomarker or a predefined institutional threshold.

Factors routinely considered during tumor board discussion included Simpson grade, tumor location, histopathological characteristics, mitotic index, Ki-67 labeling index, presence of brain invasion when documented, feasibility of salvage surgery or radiotherapy at recurrence, patient age, comorbidities, expected RT-related morbidity, and patient preference. The Ki-67 labeling index was available in routine pathology reports and was considered a supplementary proliferation marker, although no universal Ki-67 threshold was used to mandate adjuvant RT.

Brain invasion was recorded from the final pathology report as present, absent, or not documented. Because status was not documented in 12 of the 56 patients in the primary cohort, brain invasion was summarized descriptively and evaluated in a documented-case sensitivity analysis rather than included in the main multivariable models.

### 2.6. Adjuvant Radiotherapy Decision and Group Assignment

Patients were stratified into two groups according to the postoperative management strategy selected by the multidisciplinary tumor board. The adjuvant RT group included patients who received postoperative radiotherapy within the first 3 months after surgery. The observation group included patients managed without upfront adjuvant RT and followed with serial contrast-enhanced MRI.

Because treatment allocation was based on clinical judgment rather than randomization, the comparison between groups was interpreted as an analysis of real-world multidisciplinary decision-making patterns rather than as a causal assessment of treatment efficacy. Accordingly, recurrence, progression-free survival, overall survival, and other treatment-effect outcomes were not compared between the RT and observation groups.

### 2.7. Candidate Determinants

Candidate variables evaluated for their association with observed adjuvant RT use were age, sex, preoperative neutrophil-to-lymphocyte ratio (NLR), tumor size (maximum diameter on preoperative MRI), anatomical location (convexity versus non-convexity), Ki-67 labeling index, and mitotic index (mitoses/10 HPF). Non-convexity location was retained descriptively but excluded from the adjusted models because of sparse representation.

Histological subtype and presentation status (primary versus recurrent/residual disease) were recorded as descriptive cohort characteristics. Primary presentation referred to surgery following the initial diagnosis. These variables were not included in the regression models because few patients had histological subtypes other than atypical meningioma, and only one patient in the primary analytic cohort had recurrent/residual disease.

### 2.8. Study Objective and Outcome

The primary objective was to examine associations between the candidate clinical, radiological, and pathological variables and observed adjuvant RT use after Simpson grade I resection.

The primary outcome was receipt of adjuvant RT within the first 3 months after surgery, recorded as a binary variable. This outcome reflected observed postoperative treatment allocation rather than treatment efficacy or patient prognosis.

### 2.9. Statistical Analysis

The distributions of continuous variables were assessed graphically and using the Shapiro–Wilk test. These variables are reported as medians [interquartile ranges, IQRs]. Categorical variables are reported as *n* (%). Between-group comparisons in the Simpson grade I cohort used the Mann–Whitney U test for continuous variables and Fisher’s exact test for categorical variables. Because postoperative management was determined by multidisciplinary clinical judgment rather than random allocation, baseline comparisons were not interpreted as tests of group comparability, and no causal inference regarding RT efficacy was drawn from descriptive differences between management groups. The relationship between Ki-67 and mitotic index was assessed using Spearman’s rank correlation. Ki-67 values according to documented brain-invasion status were additionally compared using the Mann–Whitney U test.

Univariable and adjusted associations with observed adjuvant RT use were estimated using Firth-penalized logistic regression to reduce separation and small-sample bias [14]. Separate adjusted models included age, sex, log2-transformed NLR, tumor size, and either Ki-67 or mitotic index. Ki-67 and mitotic index were not entered together because of their correlation. Non-convexity location was excluded from the adjusted models because only six patients were in this category. Firth penalization does not overcome the limited information associated with 24 RT events; adjusted estimates were therefore interpreted as exploratory. Odds ratios (ORs) are reported with 95% confidence intervals (CIs).

The apparent ability of Ki-67 and mitotic index to discriminate between patients who did and did not receive adjuvant RT was summarized using receiver operating characteristic (ROC) analysis, area under the curve (AUC) estimates with 95% CIs, and paired DeLong comparison [15]. Sensitivity and specificity at the apparent Youden-optimal threshold describe agreement with observed allocation rather than diagnostic accuracy against an independent reference standard; likelihood ratios were therefore not calculated. Ki-67 values were recorded as integer percentages. Threshold stability was assessed by repeating threshold selection in 5000 bootstrap samples.

The incremental contribution of tumor size beyond Ki-67 was evaluated using 1000 bootstrap samples. The entire Firth model was re-estimated in each bootstrap sample and evaluated in the bootstrap and original samples. Apparent and optimism-corrected AUC and Brier score, together with the bootstrap-estimated calibration slope, were reported for the Ki-67-only and Ki-67-plus-tumor-size models.

A sensitivity analysis was performed among the 44 patients with documented brain-invasion status using a parsimonious Firth model containing Ki-67 and brain invasion. This analysis was intended to assess potential confounding and was not interpreted as a treatment-effect model. No comparative analysis of recurrence, progression-free survival, overall survival, or mortality was performed because assessment of the therapeutic effectiveness of adjuvant RT was not an objective of the present study.

All statistical tests were two-sided, and *p* < 0.05 was considered statistically significant. Analyses were conducted using jamovi version 2.7.38 and R version 4.3.3; the logistf and pROC packages were used for Firth regression and ROC analyses, respectively.

Relevant principles of the TRIPOD framework were used to guide transparent reporting of the multivariable discrimination and internal-validation analyses [16]. Because the present study was designed primarily to characterize determinants of an observed clinical treatment decision rather than to develop a validated prognostic or diagnostic prediction model, TRIPOD was used as a reporting framework rather than as a claim of full prediction-model validation.

## 3. Results

### 3.1. Cohort Characteristics and Adjuvant Radiotherapy Use

The full cohort comprised 65 patients with WHO grade 2 meningioma. Of these, 56 patients underwent Simpson grade I resection and constituted the primary analytic cohort. Adjuvant RT was administered to 24 patients (42.9%), whereas 32 patients (57.1%) were managed without upfront RT. The remaining nine patients underwent Simpson grade II or III resection, and all received adjuvant RT. Because Simpson grade was therefore completely associated with RT allocation among these patients, the primary treatment-decision analyses were restricted to the homogeneous Simpson grade I cohort.

Clinical, anatomical, and pathological characteristics of the primary analytic cohort according to adjuvant RT use are summarized in Table 1. The recorded histological diagnoses were atypical meningioma in 50 patients (89.3%), clear cell meningioma in five (8.9%), and transitional meningioma classified as WHO grade 2 in one (1.8%). Fifty-five patients (98.2%) underwent surgery following their initial diagnosis, whereas one (1.8%) presented with recurrent/residual disease. The latter patient received adjuvant RT. Histological subtype and presentation status are summarized by treatment group in Table 1.

Patients receiving adjuvant RT had substantially higher proliferative indices than those managed without RT. The median Ki-67 labeling index was 20.0% [15.0–30.0] in the RT group compared with 8.5% [8.0–10.0] in the observation group (*p* < 0.001). Similarly, the median mitotic index was 8.0 [5.8–12.0] versus 4.0 [2.8–5.2] mitoses/10 HPF, respectively (*p* < 0.001). Age, sex, NLR, tumor size, and non-convexity location did not differ significantly between the two management groups. Among the 44 patients with documented brain-invasion status, RT was administered to 11 of 16 patients (68.8%) with brain invasion and 8 of 28 patients (28.6%) without brain invasion (Fisher’s exact *p* = 0.013).

### 3.2. Determinants of Observed Adjuvant RT Use

Univariable and Firth-penalized multivariable logistic regression analyses are presented in Table 2. Ki-67 and mitotic index were evaluated in separate adjusted models because of their correlation.

Higher Ki-67 labeling index was associated with observed adjuvant RT use. Each 5-percentage-point increase corresponded to a univariable OR of 5.14 (95% CI 2.05–12.90; *p* < 0.001) and an adjusted OR of 7.10 (95% CI 2.20–22.94; *p* = 0.001) after adjustment for age, sex, NLR, and tumor size. The wide confidence interval reflects the limited information available for multivariable estimation.

The mitotic index was also associated with observed RT allocation. The OR per 5 mitoses/10 HPF was 4.36 (95% CI 1.69–11.26; *p* = 0.002) in univariable analysis and 5.81 (95% CI 2.01–16.75; *p* = 0.001) in the adjusted model.

Neither age nor sex showed an association with observed RT allocation in the adjusted models. Estimates for NLR and tumor size were imprecise and varied modestly between the two biomarker models; they were not interpreted as established determinants of allocation.

### 3.3. Discriminatory Performance of Ki-67 and Mitotic Index

**Figure 1:**
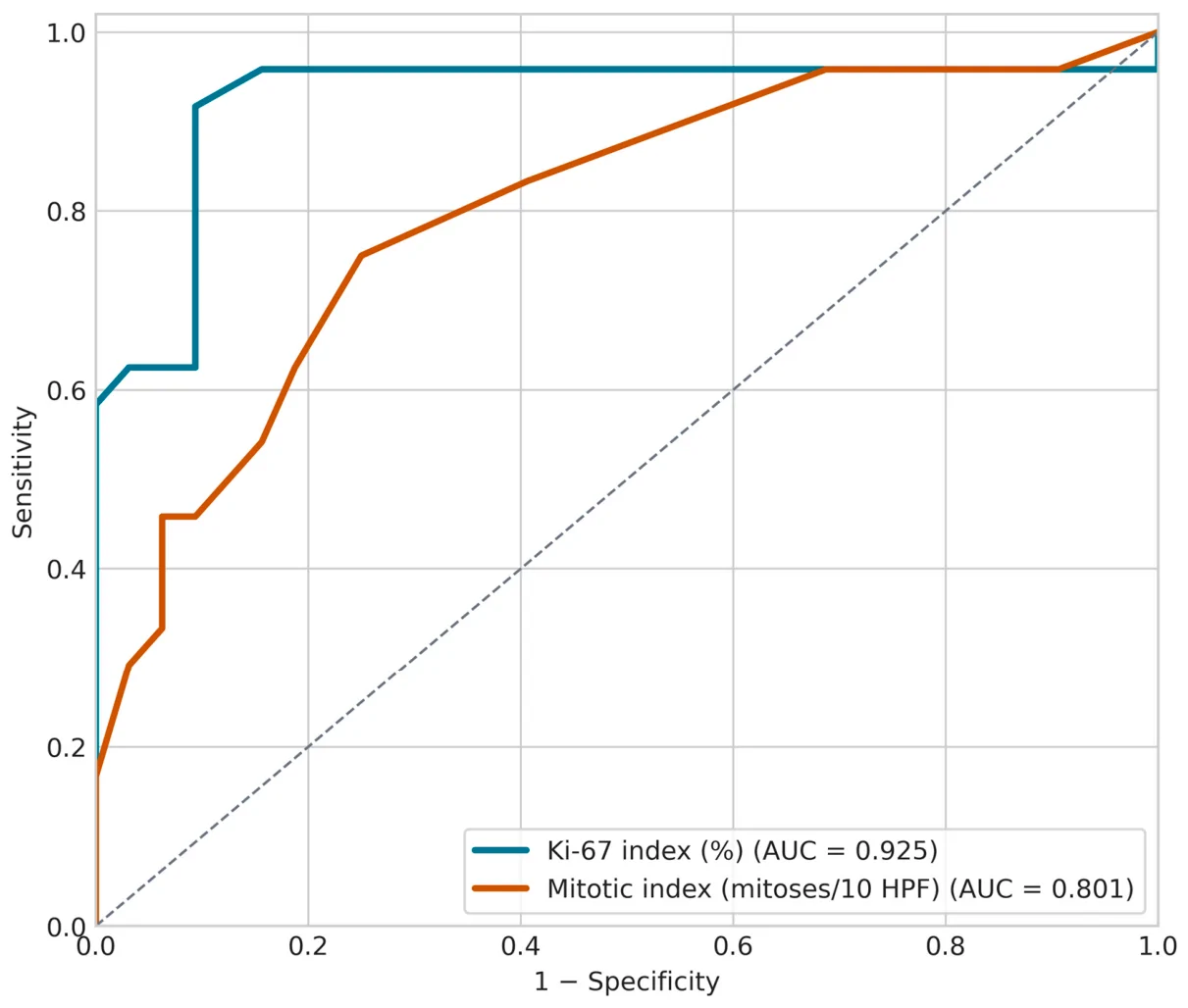
ROC curves of Ki-67 and mitotic index for discriminating observed adjuvant radiotherapy use after Simpson grade I resection. The diagonal dashed line represents chance-level discrimination (AUC = 0.5).

The discriminatory performance of Ki-67 and mitotic index for observed adjuvant RT allocation is presented in Table 3 and Figure 1.

Ki-67 had an apparent AUC of 0.925 (95% CI 0.837–1.000). The apparent Youden-optimal classification rule was Ki-67 ≥ 14%, yielding 91.7% sensitivity and 90.6% specificity for observed RT allocation. These measures describe agreement with allocation and do not represent diagnostic performance against an independent clinical reference standard.

The mitotic index had an apparent AUC of 0.801 (95% CI 0.682–0.920). A threshold of ≥6 mitoses/10 HPF yielded 75.0% sensitivity and 75.0% specificity for observed RT allocation. Ki-67 had a higher apparent AUC than mitotic index (paired DeLong *p* = 0.008).

The 14% threshold was not stable across bootstrap resamples: thresholds of 12%, 14%, and 15% were selected in 33.4%, 41.9%, and 23.1% of 5000 samples, respectively. The threshold was therefore interpreted as a cohort-specific description of allocation rather than a clinical decision point.

### 3.4. Incremental Value of Tumor Size

Bootstrap internal-validation results are summarized in Table 4. For Ki-67 alone, the apparent and optimism-corrected AUCs were 0.925 and 0.925; the corresponding Brier scores were 0.093 and 0.103, and the bootstrap calibration slope was 0.980. The near-zero AUC optimism for a single fixed marker does not validate the selected threshold.

**Figure 2:**
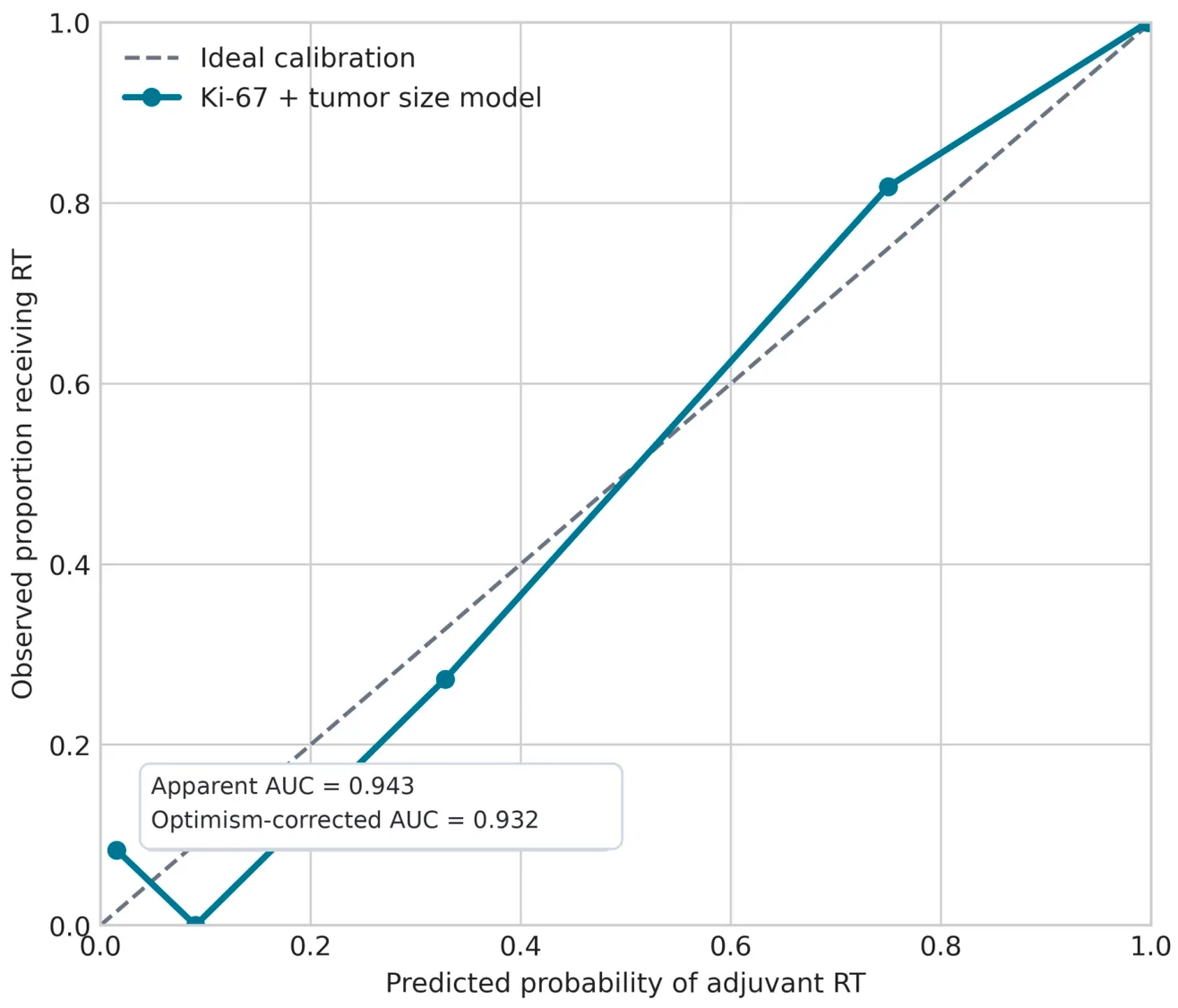
Calibration plot of the final Ki-67 plus tumor-size model for observed adjuvant radiotherapy use after Simpson grade I resection.

Adding tumor size increased the apparent AUC to 0.943, whereas the optimism-corrected AUC was 0.932. The optimism-corrected Brier score was 0.101, and the bootstrap calibration slope was 0.897. This modest increment was interpreted as exploratory. The calibration plot of the Ki-67 plus tumor-size model is shown in Figure 2.

### 3.5. Brain-Invasion Sensitivity Analysis

The results of the sensitivity analysis incorporating documented brain-invasion status are presented in Table 5. Among patients with documented brain-invasion status, Ki-67 values were higher in those with brain invasion than in those without brain invasion (median 29.0% [IQR 14.3–32.5] vs. 9.5% [IQR 8.0–14.3]; *p* < 0.001). In the two-predictor Firth sensitivity model, Ki-67 remained associated with observed RT allocation (OR 4.53 per 5-percentage-point increase, 95% CI 1.77–11.63; *p* = 0.002). The brain-invasion estimate was highly imprecise and changed direction after Ki-67 adjustment (OR 0.49, 95% CI 0.05–5.24; *p* = 0.558), indicating substantial overlap between the two pathological variables. The documented-case sensitivity analysis does not eliminate residual confounding or establish independent causal effects for Ki-67 or brain invasion.

The sensitivity analysis was exploratory and was not designed to identify indications for RT. The distribution of recorded Ki-67 values according to observed adjuvant RT allocation is presented in Table 6.

A Ki-67 threshold of ≥14% and a boundary of >13% classify the same patients because no recorded Ki-67 value was 13%.

Bootstrap variation among thresholds of 12%, 14%, and 15% demonstrates that the numerical boundary is not stable.

## 4. Discussion

In this real-world cohort of patients with WHO grade 2 meningioma undergoing Simpson grade I resection, Ki-67 and mitotic index were associated with observed RT allocation after adjustment for age, sex, NLR, and tumor size. Non-convexity location was not included in the adjusted models because only six patients were in this category. Because Ki-67 and mitotic index were available to the tumor board, these associations describe alignment with the observed allocation process and should not be interpreted as evidence that either marker identifies patients who benefit from RT.

The apparent Ki-67 threshold of 14% should be interpreted cautiously. Ki-67 was recorded as integer percentages, and bootstrap resampling frequently selected 12% or 15% instead. Proximity to previously reported prognostic cutoffs does not validate this value as a treatment threshold, particularly because the tumor board had access to Ki-67 when making treatment decisions.

Measurement variability adds further uncertainty. Interobserver agreement for Ki-67 was assessed in only 20 cases, yielding an ICC of 0.76. This estimate does not establish that measurements near 14% would consistently fall on the same side of the threshold across observers. Although disagreements were resolved through joint slide review, consensus assessment does not eliminate variability across observers or institutions. Bootstrap resampling of the recorded values assesses threshold instability due to sampling but does not directly capture interobserver measurement error. The strong association between Ki-67 and observed RT allocation therefore raises the question of how sensitive treatment decisions may be to variation in Ki-67 assessment. These data cannot establish whether the observed reliance on Ki-67 was clinically appropriate, and the derived threshold should not be used as a stand-alone treatment criterion.

The mitotic index also showed a strong association with RT allocation but demonstrated lower discriminatory performance than Ki-67 (AUC 0.801 vs. 0.925; DeLong *p* = 0.008). One possible explanation is that mitotic activity is incorporated directly into the diagnostic framework for WHO grade 2 meningioma, whereas Ki-67 provides a continuous measure of proliferative activity that may capture additional biological heterogeneity within a population already defined by histological grade [8]. Previous studies have similarly demonstrated prognostic value for mitotic count, while some series have suggested that Ki-67 provides a stronger or more consistent association with recurrence-related outcomes [11,12]. In this context, the present findings support the potential value of Ki-67 as a complementary marker in postoperative multidisciplinary risk assessment rather than as a replacement for established histopathological criteria. The regression, ROC, and bootstrap analyses are related evaluations of the same observations and should not be regarded as independent replications.

Adding tumor size to Ki-67 produced only a modest increase in discrimination after bootstrap correction. Non-convexity location was reported descriptively and excluded from the adjusted models because only six patients were in this category. The small number of RT events limited the precision of multivariable estimates, and Firth penalization could not compensate for this limited information.

Brain invasion was associated with RT allocation in the unadjusted documented-case comparison and was also associated with higher Ki-67 values. When both variables were included in a parsimonious sensitivity model, the Ki-67 association persisted, whereas the brain-invasion estimate became highly imprecise and changed direction. This instability indicates substantial overlap between the pathological variables and prevents reliable separation of their independent contributions.

An important methodological feature of this study was the restriction of the primary analysis to patients undergoing Simpson grade I resection. In the full cohort, all patients with Simpson grades II–III resection received adjuvant RT, creating complete separation between extent of resection and treatment allocation. Including these patients in the adjusted models would therefore have made it difficult to distinguish the independent contribution of proliferative markers from that of the extent of resection. Restricting the primary analysis to Simpson grade I resection minimized this specific source of confounding and allowed treatment-selection determinants to be evaluated within a more homogeneous surgical cohort. This design choice does not eliminate other sources of confounding, but it strengthens the internal interpretability of the observed associations.

The present findings should be interpreted within the broader uncertainty surrounding adjuvant RT after complete resection of WHO grade 2 meningioma. The EORTC 22042-26042 and NRG Oncology RTOG 0539 studies reported favorable disease control following postoperative RT, but neither provided a randomized comparison with observation [5,6]. Retrospective studies and meta-analyses have also suggested potential benefits of adjuvant RT after gross-total resection, particularly in terms of local control and progression-free survival [17,18,19]. More recently, a large international multicenter cohort suggested that the benefit of adjuvant fractionated RT after GTR remains less clearly defined than after subtotal resection, supporting individualized postoperative decision-making [20]. The ROAM/EORTC-1308 trial was designed specifically to address this question through randomization between early RT and active surveillance [4,7]. The present study does not address treatment efficacy; instead, it characterizes the factors associated with how this unresolved clinical question is currently managed in practice.

This distinction is particularly relevant for retrospective outcome studies. Because patients selected for adjuvant RT may differ systematically from those managed with observation, any comparison of recurrence or survival outcomes is vulnerable to confounding by indication. The strong association between proliferative markers and RT allocation observed in the present cohort illustrates this problem directly. Future retrospective studies evaluating RT effectiveness should therefore account for Ki-67, mitotic activity, extent of resection, and other treatment-selection variables through appropriate stratification, multivariable adjustment, propensity-based methods, or other causal-inference approaches.

The evolving molecular classification of meningioma further highlights the limitations of relying solely on conventional histopathological markers. DNA methylation-based classification, integrated molecular-morphological models, and molecular alterations such as TERT promoter mutation and CDKN2A/B homozygous deletion have demonstrated prognostic relevance and may refine risk stratification beyond conventional histological grading [21,22,23,24,25]. These molecular and epigenetic data were not available in the present cohort. It therefore remains uncertain whether the Ki-67-dominant treatment-selection pattern identified here would persist if contemporary molecular risk markers were routinely incorporated into multidisciplinary postoperative decision-making.

This study has several limitations. The modest primary cohort and 24 RT events limit multivariable precision; Firth penalization reduces separation and small-sample bias but does not overcome limited information. The ROC measures quantify agreement with observed allocation rather than diagnostic accuracy for treatment benefit. The Ki-67 threshold was data-derived and unstable under bootstrap resampling. Brain-invasion status was not documented in 12 patients, and the documented-case sensitivity model remained imprecise. Internal validation cannot substitute for external validation. One patient had recurrent/residual disease and received adjuvant RT. The contribution of presentation status to treatment allocation could not be reliably estimated from this single case.

Ki-67 was the measured variable most strongly associated with observed RT allocation. Agreement among the related analyses reflects internal consistency within this dataset rather than independent confirmation or validation of a clinical decision rule.

This study should be regarded as hypothesis-generating. Larger multicenter cohorts are needed to determine whether the observed associations reflect broader treatment-allocation patterns or institution-specific practice. Future studies should use a standardized Ki-67 assessment and a prespecified external-validation protocol in which the exploratory 14% threshold is evaluated without re-optimization in an independent cohort. Such validation would assess its reproducibility in describing observed RT allocation. Establishing whether this threshold identifies patients who benefit from RT would require a separate evaluation of clinical outcomes and treatment effects.

## 5. Conclusions

In patients with WHO grade 2 meningioma undergoing Simpson grade I resection, proliferative markers—particularly Ki-67—were strongly associated with observed adjuvant RT allocation. The data-derived 14% Ki-67 threshold was unstable under bootstrap resampling and should be interpreted only as a cohort-specific description of allocation, not as a validated threshold for recommending RT. The analyses characterize observed treatment-selection patterns and do not evaluate treatment efficacy.

## Figures and Tables

**Table 1:** Clinical, anatomical, and pathological characteristics of patients undergoing Simpson grade I resection according to adjuvant radiotherapy use.

Characteristic	No Adjuvant RT (*n* = 32)	Adjuvant RT (*n* = 24)	*p*
Age, years	61.0 [45.5–72.0]	65.5 [45.2–70.5]	0.855
Neutrophil-to-lymphocyte ratio	2.8 [1.8–7.1]	2.7 [1.8–5.1]	0.412
Ki-67 index, %	8.5 [8.0–10.0]	20.0 [15.0–30.0]	<0.001
Mitotic index, mitoses/10 HPF	4.0 [2.8–5.2]	8.0 [5.8–12.0]	<0.001
Tumor size, mm	39.5 [30.0–55.0]	38.0 [18.8–48.5]	0.353
Female sex, *n* (%)	13 (40.6)	13 (54.2)	0.418
Male sex, *n* (%)	19 (59.4)	11 (45.8)	
Non-convexity location, *n* (%)	4 (12.5)	2 (8.3)	0.691
Brain invasion absent, *n* (%)	20 (62.5)	8 (33.3)	0.013
Brain invasion present, *n* (%)	5 (15.6)	11 (45.8)	
Brain invasion not documented, *n* (%)	7 (21.9)	5 (20.8)	
Histological subtype, *n* (%)			
Atypical meningioma	29 (90.6)	21 (87.5)	
Clear cell meningioma	3 (9.4)	2 (8.3)	
Transitional meningioma, WHO grade 2	0 (0.0)	1 (4.2)	
Presentation, *n* (%)			
Primary	32 (100.0)	23 (95.8)	
Recurrent/residual	0 (0.0)	1 (4.2)	

Continuous variables are presented as median [interquartile range] and were compared using the Mann–Whitney U test. Categorical variables are presented as *n* (%) and were compared using Fisher’s exact test. HPF: high-power field; NLR: neutrophil-to-lymphocyte ratio; RT: radiotherapy. The brain-invasion *p* value compares present versus absent status among the 44 patients with documented brain-invasion status. Histological subtype and presentation status are reported descriptively because of sparse category counts; no hypothesis tests were performed for these variables.

**Table 2:** Univariable and adjusted Firth-penalized logistic regression analyses for observed adjuvant radiotherapy use after Simpson grade I resection.

Candidate Variable	Univariable OR (95% CI)	*p*	Adjusted Ki-67 Model OR (95% CI)	*p*	Adjusted Mitotic-Index Model OR (95% CI)	*p*
Age, per 10 years	1.04 (0.76–1.41)	0.815	1.08 (0.60–1.93)	0.797	1.15 (0.76–1.72)	0.514
Male vs. female	0.59 (0.20–1.72)	0.332	1.07 (0.18–6.28)	0.937	0.55 (0.15–2.05)	0.372
NLR, per doubling	0.87 (0.59–1.29)	0.488	0.50 (0.24–1.03)	0.062	0.59 (0.33–1.03)	0.064
Tumor size, per 10 mm	0.87 (0.65–1.16)	0.327	0.59 (0.33–1.07)	0.082	0.69 (0.47–1.02)	0.065
Ki-67, per 5 percentage points	5.14 (2.05–12.90)	<0.001	7.10 (2.20–22.94)	0.001	—	—
Mitotic index, per 5 mitoses/10 HPF	4.36 (1.69–11.26)	0.002	—	—	5.81 (2.01–16.75)	0.001

Adjusted models included age, sex, log2-transformed NLR, tumor size, and either Ki-67 or mitotic index. Non-convexity location was excluded because only six patients were in this category. CI: confidence interval; HPF: high-power field; NLR: neutrophil-to-lymphocyte ratio; OR: odds ratio. Ki-67 and mitotic index were positively correlated (Spearman ρ = 0.657, *p* < 0.001); their results were therefore regarded as related analyses rather than independent confirmation. An em dash (—) indicates that the variable was not included in the corresponding model.

**Table 3:** ROC analysis of Ki-67 and mitotic index for observed adjuvant radiotherapy allocation after Simpson grade I resection.

Marker	AUC (95% CI)	Apparent Allocation Threshold	Sensitivity, %	Specificity, %
Ki-67 index, %	0.925 (0.837–1.000)	≥14.0	91.7	90.6
Mitotic index, mitoses/10 HPF	0.801 (0.682–0.920)	≥6.0	75.0	75.0

AUC: area under the receiver operating characteristic curve; CI: confidence interval; HPF: high-power field. Sensitivity and specificity describe agreement with observed RT allocation and should not be interpreted as diagnostic accuracy for RT benefit.

**Table 4:** Bootstrap internal validation of Ki-67-based models for observed adjuvant radiotherapy allocation.

Model	Predictors	Apparent AUC	Optimism-Corrected AUC	Apparent Brier Score	Optimism-Corrected Brier Score	Bootstrap Calibration Slope
Model 1	Ki-67	0.925	0.925	0.093	0.103	0.980
Model 2	Ki-67 + tumor size	0.943	0.932	0.086	0.101	0.897

**Table 5:** Sensitivity analysis incorporating documented brain-invasion status.

Variable	OR (95% CI)	*p*
Ki-67, per 5 percentage points	4.53 (1.77–11.63)	0.002
Brain invasion, present vs. absent	0.49 (0.05–5.24)	0.558

The model included the 44 patients with documented brain-invasion status. Estimates were obtained using Firth-penalized logistic regression. CI: confidence interval; OR: odds ratio.

**Table 6:** Distribution of recorded Ki-67 values according to observed adjuvant radiotherapy allocation.

Recorded Ki-67, %	No Adjuvant RT, *n*	Adjuvant RT, *n*
1	0	1
3	4	0
4	1	0
5	1	0
6	1	0
8	9	0
9	2	0
10	9	0
12	2	1
14	0	1
15	0	6
16	1	0
17	1	0
18	1	1
20	0	3
24	0	1
28	0	2
30	0	3
32	0	1
34	0	1
35	0	2
60	0	1

Ki-67 values were recorded as integer percentages; the complete distribution is presented to show measurement granularity around the selected boundary.

## Data Availability

The data supporting the findings of this study are available from the corresponding author upon reasonable request, subject to institutional and ethical restrictions.
